# The effect of a multidisciplinary lifestyle program for patients with rheumatoid arthritis, an increased risk for rheumatoid arthritis or with metabolic syndrome-associated osteoarthritis: the “Plants for Joints” randomized controlled trial protocol

**DOI:** 10.1186/s13063-021-05682-y

**Published:** 2021-10-18

**Authors:** Wendy Walrabenstein, Marike van der Leeden, Peter Weijs, Henriët van Middendorp, Carlijn Wagenaar, Johanna Maria van Dongen, Max Nieuwdorp, Catharina Sophia de Jonge, Laurette van Boheemen, Dirkjan van Schaardenburg

**Affiliations:** 1 Amsterdam Rheumatology and Immunology Center, Reade, Dr. Jan van Breemenstraat 2, 1056 AB Amsterdam, The Netherlands; 2 Department of Rheumatology and Clinical Immunology, Amsterdam UMC, University of Amsterdam, Meibergdreef 9, 1105 AZ Amsterdam, The Netherlands; 3 Department of Rehabilitation Medicine, Amsterdam UMC, VU University, De Boelelaan, 1117 1081 HV Amsterdam, The Netherlands; 4 Amsterdam Public Health Research Institute, De Boelelaan, 1085 1081 HV Amsterdam, The Netherlands; 5 Amsterdam University of Applied Sciences, Dokter Meurerlaan 8, 1067 SM Amsterdam, The Netherlands; 6 Department of Nutrition & Dietetics, Amsterdam University Medical Centers, VU University, De Boelelaan 1117, 1081 HV Amsterdam, The Netherlands; 7 Leiden University, Institute of Psychology, Health, Medical, & Neuropsychology unit, Leiden, The Netherlands; 8 Department of Health Sciences, Faculty of Science, Vrije Universiteit Amsterdam, De Boelelaan, 1085 1081 HV Amsterdam, The Netherlands; 9 Department of Internal Medicine, Amsterdam UMC, University of Amsterdam, Meibergdreef 9, 1105 AZ Amsterdam, The Netherlands; 10 Departments of Radiology and Nuclear Medicine & Gastroenterology Endocrinology Metabolism, Amsterdam UMC, University of Amsterdam, Meibergdreef 9, 1105 AZ Amsterdam, The Netherlands

## Abstract

Low-grade inflammation and metabolic syndrome are seen in many chronic diseases, including rheumatoid arthritis (RA) and osteoarthritis (OA). Lifestyle interventions which combine different non-pharmacological therapies have shown synergizing effects in improving outcomes in patients with other chronic diseases or increased risk thereof, especially cardiovascular disease. For RA and metabolic syndrome-associated OA (MSOA), whole food plant-based diets (WFPDs) have shown promising results. A WFPD, however, had not yet been combined with other lifestyle interventions for RA and OA patients. In this protocol paper, we therefore present Plants for Joints, a multidisciplinary lifestyle program, based on a WFPD, exercise, and stress management. The objective is to study the effect of this program on disease activity in patients with RA (randomized controlled trial [RCT] 1), on a risk score for developing RA in patients with anti-citrullinated protein antibody (ACPA) positive arthralgia (RCT 2) and on pain, stiffness, and function in patients with MSOA (RCT 3), all in comparison with usual care.

We designed three 16-week observer-blind RCTs with a waiting-list control group for patients with RA with low to moderate disease activity (2.6 ≤ Disease Activity Score [DAS28] ≤ 5.1, RCT 1, *n* = 80), for patients at risk for RA, defined by ACPA-positive arthralgia (RCT 2, *n* = 16) and for patients with metabolic syndrome and OA in the knee and/or hip (RCT 3, *n* = 80). After personal counseling on diet and exercise, participants join 10 group meetings with 6–12 other patients to receive theoretical and practical training on a WFPD, exercise, and stress management, while medication remains unchanged. The waiting-list control group receives usual care, while entering the program after the RCT. Primary outcomes are: difference in mean change between intervention and control groups within 16 weeks for the DAS28 in RA patients (RCT 1), the RA-risk score for ACPA positive arthralgia patients (RCT 2), and the Western Ontario and McMaster Universities Arthritis Index (WOMAC) score for MSOA patients (RCT 3). Continued adherence to the lifestyle program is measured in a two-year observational extension study.

**Supplementary Information:**

The online version contains supplementary material available at 10.1186/s13063-021-05682-y.

## Introduction

The development of rheumatoid arthritis (RA) and osteoarthritis (OA) have been linked to diet and obesity [1–8]. Studies indicate fasting, a Mediterranean diet, as well as a whole food plant-based diet (WFPD) can lower disease activity in patients with RA [9–14]. In OA, hypocaloric and WFPDs showed favorable results, while the effect of a hypocaloric diet in combination with exercise was superior, suggesting synergies for multidisciplinary interventions [15–18].

For exercise, it has been shown that physical activity is associated with a lower risk of RA [19]. In patients with RA, long-term high-intensity exercise is effective in improving functional and emotional status [20]. Routinely combining aerobic exercise with muscle strength training (similar to the Dutch Exercise Guideline) is recommended for patients with RA [21]. Additionally, for patients with OA in the hip and/or the knee, exercise improves physical function and lowers pain [16, 17].

For both RA patients and patients at increased risk for RA, the heart rate is controlled increasingly by the sympathetic nervous system in contrast to the parasympathetic nervous system. This results in a lower heart rate variability suggesting a higher level of stress [22, 23]. The onset of RA has also been linked to stress [6, 7, 24], and both Mindfulness Based Stress Reduction (MBSR) as well as internet-based cognitive-behavioral therapy have shown favorable outcomes, especially with regard to general wellbeing [25, 26]. For OA, a small pilot study showed a significant reduction of pain and improvement of function in patients who followed an 8-week meditation program. In OA patients, higher baseline “mindfulness” scores were also associated with a better response to exercise than patients with lower baseline mindfulness, suggesting again the synergistic effects of combining more disciplines within one intervention [27].

A multidisciplinary program based on a WFPD, exercise, and stress management showed favorable results for patients with coronary artery disease [28]. Although the separate components of this multidisciplinary lifestyle intervention were found to be beneficial for RA and OA patients, no studies have yet been conducted to investigate the effect of an integrated program for RA or OA.

The presence of low-grade inflammation is a feature of many chronic diseases including RA and OA [29]. In RA, a chronic autoimmune arthritis condition, the presence of low-grade inflammation—possibly intermediated by microbiome dysbiosis—may cause the breakdown of immune tolerance [29, 30]. Support for the mucosal origin of RA comes from the observation that part of the circulating RA-associated autoantibodies rheumatoid factor (RF) and anti-citrullinated protein antibodies (ACPA) are of mucosal origin [30]. Additionally, compared to healthy individuals, an altered, or dysbiotic, microbiome is seen in RA and its preclinical stage, with, among others, an overrepresentation of *Lactobacillus salivarius* and *Prevotella copri* [31]. As the microbiome is affected among others by diet, exercise, and stress, these lifestyle factors may therefore contribute to the pathogenesis of RA at mucosal sites [30–33].

Low-grade inflammation is also associated with metabolic syndrome, a combination of risk factors such as high fat mass (waist circumference), high blood pressure, high low-density lipoprotein (LDL), low high-density lipoprotein (HDL), high triglycerides, and high fasting glucose, which are very common in patients with OA [29, 34]. Levels of inflammatory mediators are higher in people with visceral adiposity, which may mediate the relationship between OA and obesity [35]. This has resulted in the denomination of metabolic syndrome-associated osteoarthritis (MSOA) as a specific form of OA [36]. MSOA is a chronic condition with limited treatment options (analgesics, nonsteroidal anti-inflammatory drugs (NSAIDs), and intra-articular glucocorticoids), and although guidelines for the treatment of OA also recommend exercise treatment, weight loss, and mental health interventions, they also emphasize the need for more studies, especially regarding multidisciplinary interventions.

The systemic impact of low-grade inflammation, microbiome dysbiosis, and increased (mostly visceral) fat mass also explains the frequent occurrence of comorbidities, such as diabetes and cardiovascular disease in patients with OA and RA [34, 37].

### Research objective

The objective of Plants for Joints is to study the effects of a multidisciplinary lifestyle program in patients with (risk for) RA and in patients with MSOA. The program consists of a WFPD, exercise, and stress management and aims to lower disease activity in patients with RA (randomized controlled trial [RCT] 1), to lower the risk of RA [38] in patients with ACPA-positive arthralgia (RCT 2), or to improve the scores for pain, stiffness and function in patients with MSOA (RCT 3), all in comparison to usual care. An observational extension study is added to investigate adherence.

## Design and methods

### Design

We designed three 16-week observer-blind RCTs with a waiting-list control group for patients with RA (RCT1), for patients at risk for RA, defined by ACPA-positive arthralgia (RCT 2) and for patients with MSOA (RCT 3) [39]. All participants receive the program, either directly or after participation in the control group.

Medication for RA is kept stable during the RCT (16 weeks for the intervention group, 32 weeks for the control group) whenever possible. DMARD changes (in RA patients) and intercurrent corticosteroid administration is considered a protocol deviation and the reason for the change will be registered.

In a 2-year extension study, starting at the end of the 16-week lifestyle program, all patients receive continued online support and 6 additional, thematic and facultative meetings. During the extension study, we will investigate adherence to the lifestyle program in relation to long-term effects and success factors for and barriers to changing behavior.

Study visits are at baseline, 8 and 16 weeks during the RCT, at 8 and 16 weeks for the waiting-list control group after entering the intervention, and every 6 months during the extension period.

**Fig. 1:**
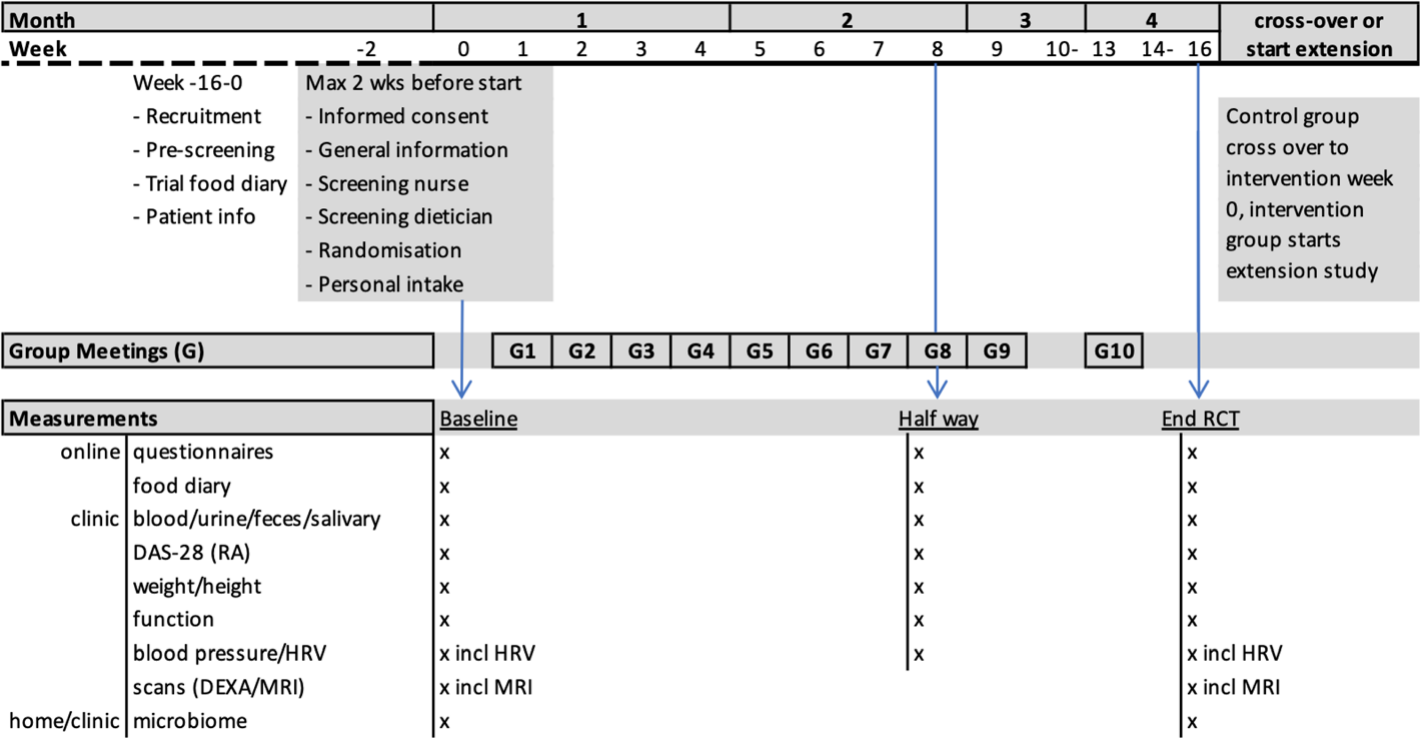
Schedule of intervention and measurements during the randomized controlled trial (RCT). DAS28, disease activity score based on 28 joints; RA, rheumatoid arthritis; HRV, heart rate variability; DEXA, dual-energy x-ray absorptiometry; MRI, magnetic resonance imaging

**Fig. 2:**
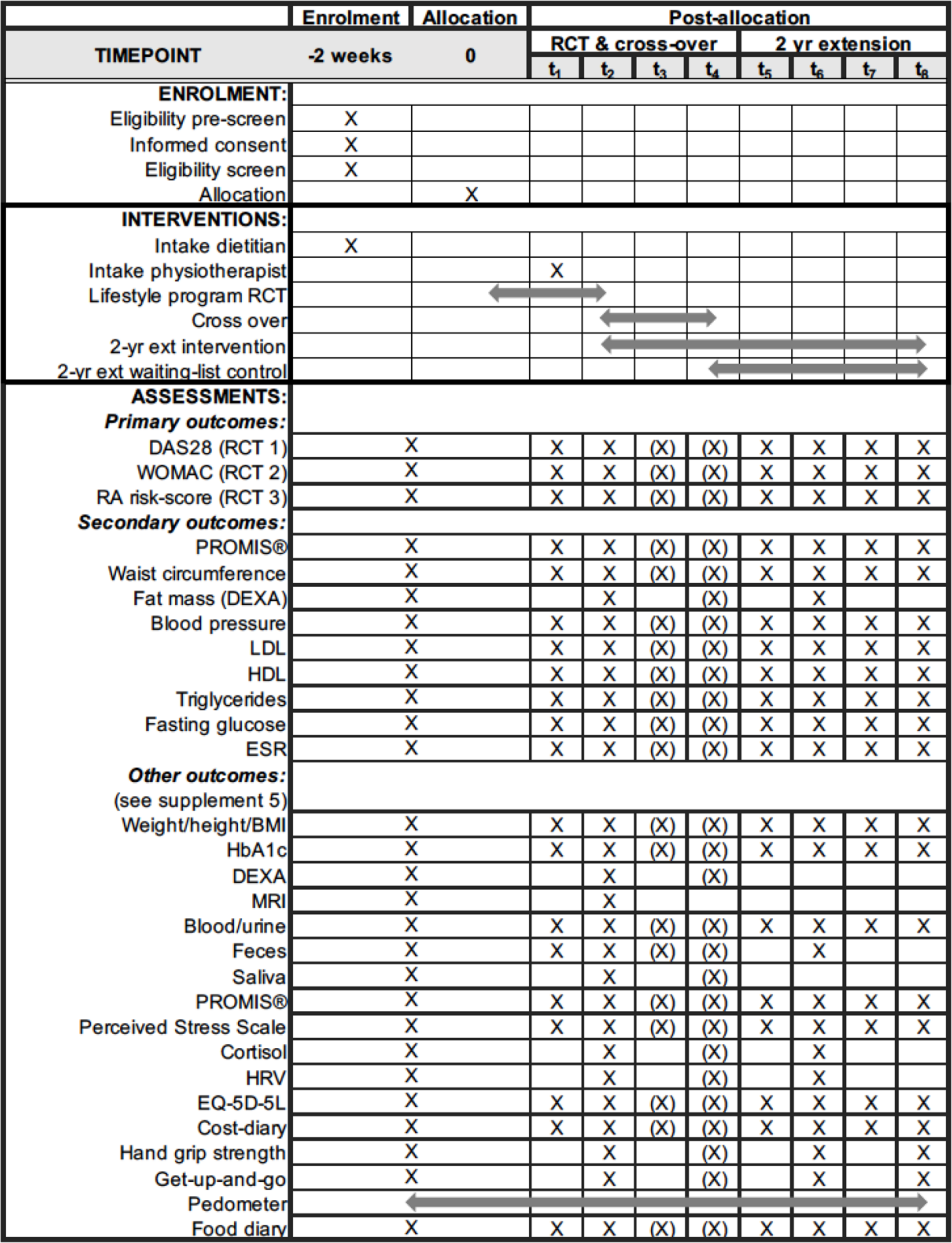
SPIRIT figure with phases of the study and data collection time points. (X) = measurement only for waiting-list control group. RCT, randomized controlled trial; yr, year; ext, extension; DAS28, disease activity score based on 28 joints; WOMAC, Western Ontario and McMaster Universities Osteoarthritis Index; RA, rheumatoid arthritis; PROMIS®, Patient Reported Outcomes Measurement Information System; DEXA, dual-energy x-ray absorptiometry; LDL, low-density lipoprotein; HDL, high-density lipoprotein; ESR, erythrocyte sedimentation rate; HbA1c, hemoglobin A1c; MRI, magnetic resonance imaging; HRV, heart rate variability; EQ-5D-5L, EuroQol 5 dimensions 5 levels

A schedule of the intervention and the measurements during the 16-week RCT is shown in Fig. 1. Figure 2 shows a comprehensive scheme based on the SPIRIT format and includes the intervention phase for the waiting-list control group and the 2-year extension study. The SPIRIT Checklist is available as an additional file (supplement 1) [39].

### Recruitment, selection, and randomization of participants

Subjects aged ≥ 18 years are recruited through rheumatologists, rehabilitation specialists, nurses and allied health care professionals at Reade and Amsterdam UMC (The Netherlands), and other regional hospitals or health centers. The main inclusion criteria are as follows: (RCT 1) RA according to the American College of Rheumatology (ACR)/European Alliance of Associations for Rheumatology (EULAR) 2010 criteria [40] with low to moderate disease activity (2.6 ≤ DAS28 ≤ 5.1) [41] and unchanged use of disease-modifying anti-rheumatic drugs (DMARDs) for 3 months or non-use of DMARDs; (RCT 2) arthralgia and positivity for ACPA [38]; and (RCT 3) OA in hip and/or knee, diagnosed according to the clinical criteria of the ACR (without age-criterion) [42, 43] and metabolic syndrome according to the criteria defined by the National Cholesterol Education Program (NCEP) Adult Treatment Panel III (ATP III) [44]. The main exclusion criteria are as follows: insufficient e-health competencies to fill in digital questionnaires and keep an online food diary, already following a predominantly plant-based diet, underweight (BMI < 18.5 kg/m^2^), pregnancy, and unwillingness to stop smoking for at least the duration of the RCT, if applicable.

The study has a parallel trial design in which patients are randomized in a 1:1 ratio by the researchers using CASTOR (an electronic database platform for data collection, management, and storage, with strictly defined user roles and patient management procedures, which keeps an audit trail) with a variable block randomization in block sizes of 2 and 4 (not stratified).

Both patients as well as study staff are not blinded for allocation. During measurements, a research nurse or other researcher, not involved in the study and not aware of allocation or phase of the study, will execute the examination for DAS28 (physical examination of the joints). Other primary and secondary outcomes are blinded by default (automized questionnaires or laboratory markers).

### Intervention

Subjects of all three RCTs participate in the same lifestyle program in mixed groups.

The program starts with a personal intake by a dietitian, reviewing general health, dietary habits, and physical activity. With a physical therapist, personal goals for physical activity are set (and reset at the beginning of the extension period). The lifestyle program consists of 10 group meetings (with 6–12 participants) for 2–3 h each (see supplement 2). During the program theoretical education about a WFPD, exercise, and stress is combined with a practical cooking class (with partner or relevant other person, in the first meeting), relaxation exercises and physical training, supported by podcasts, videos, and at-home exercises. The control group receives usual care, is advised not to change usual behavior, and enters the program after 16 weeks.

**Fig. 3:**
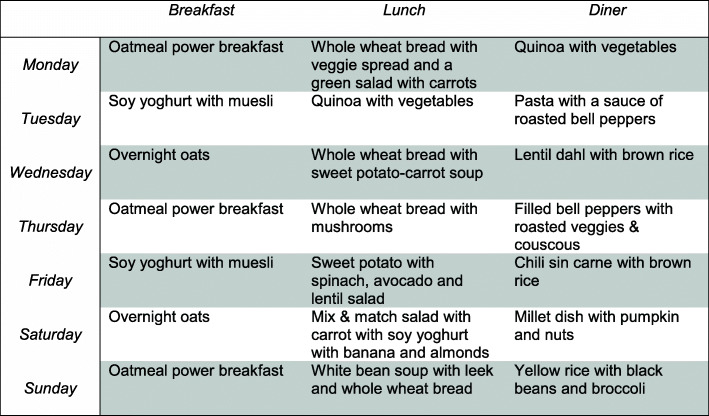
Example of a week menu

The WFPD is based on protocols by Ornish and Barnard [28, 45], although recommendations on fat are adapted to Dutch guidelines (20–35/40% of energy instead of low fat [max 10% of energy]). The proposed diet is in line with the 2015 Guidelines on Healthy Nutrition of the Health Council of the Netherlands. Subjects will be facilitated by means of fully elaborated week plans (developed by registered dietitians, in line with recommended daily allowances, see example in Fig. 3), and they will receive supplementation for vitamin B12 and D [46]. To promote weight loss, overweight and obese patients are motivated to limit meal frequency to three meals per day. The program contains a short “green fasting” protocol (see supplement 3) [9–13], as recommended by the *program Ambassadors*. The *program Ambassadors* are fifteen target group patients, who are involved in the development of the lifestyle program and the analyses. These lay experts had experienced improvement in their own health status with diet adaptations, exercise, and/or use of stress management techniques.

Recommendations for physical activity and exercise are based on the Dutch physical activity guidelines 2017 and the protocol by Ornish [28, 47]. Group exercise is focused on moderate exercise, fun, and group cohesion. Patients will be motivated to integrate physical activity in daily activities (example: walk/cycle more rather than sitting in a car or public transport) and search for possibilities to exercise in their neighborhood.

Stress management is based on protocols by De Brouwer et al. [48]. Subjects will receive psychoeducation on the effects of stress on health and on stress management, as well as guided practice and home exercises (supported by tools, such as audio/Apps) on relaxation techniques and breathing and visualization exercises, as well as coaching on sleep [48].

A specification of the subjects covered in the 10 group meetings is available in supplement 2.

During the 2-year extension study, 6 facultative meetings will be organized for participants on varying subjects from the three categories (examples: weight loss, how to stay active, self-compassion). After the 16-week intervention, medication of subjects with RA with minimal disease activity (DAS < 2.6) will be tapered by the participants’ rheumatologists, who are supplied with a standardized scheme (see supplement 4). If disease activity increases after tapering, medication will again be increased back to the previous step. For participants in the MSOA group, pain medication will be monitored.

### Measurements

#### Primary outcome measures

Main endpoints are the differences between mean changes in the intervention versus control groups from 0 to 16 weeks in:

- RCT 1: the DAS28 based on erythrocyte sedimentation rate (ESR), number of swollen joints and tender joints and general health based on the score on a visual analog scale [49] (measured by a blinded research nurse) for RA patients.
- RCT 2: the RA-risk score (combined score based on RA prevalence in first degree relatives, alcohol consumption, duration, intermittency and location of symptoms, pain, morning stiffness, self-perceived swelling of joints and antibody status) [38] in ACPA-positive arthralgia patients. If a subject develops RA during the study, the RA-risk score will be set to 13 points, the highest possible score.
- RCT 3: scores on pain, stiffness, and function, combined in the Western Ontario and McMaster Universities Osteoarthritis Index (WOMAC) index [50] for OA patients, measured by the WOMAC questionnaire [51].

Main endpoint for the 2-year extension study for all three groups is the change in adherence from 0 to 24 months, based on an adapted version of the *Lifestyle index adherence score* as developed by Ornish et al [52]:

$$ \left(\mathrm{t}+\left(\left[\mathrm{u}/6+\mathrm{v}/60\right]/2\right)+\left(\left[\mathrm{x}/5+\mathrm{y}/150\right]/2\right)+\mathrm{z}\right)/4 $$

In which *t* = attendance meetings, *u* = stress reduction activities days per week, *v* = stress reduction activities minutes per week, *x* = exercise days per week, *y* = exercise minutes per week, and *z* = adherence to diet.

Adherence to diet (*z*) is defined as ((grams of fiber per 1000 kilocalories/14) + (10/en% SFA))/2 whereby “en% SFA” is defined as the percentage of total kilocalories a day from saturated fatty acids (SFAs). In the original model by Ornish, *z* was defined by total fat and cholesterol intake. Since our protocol is not based on a low-fat WFPD, we changed these vectors into fiber and saturated fatty acids (SFAs) as indicators for a WFPD.

#### Secondary outcome measures

Secondary outcomes are the same for all groups in the three RCTs: RA, ACPA-positive arthralgia, and MSOA, unless otherwise specified. Quality of life-related outcomes measured are self-reported physical, mental and, social health, using the validated Dutch-Flemish Patient Reported Outcomes Measurement Information System (PROMIS®) [53]. In order to select the most important secondary patient reported outcomes, we consulted the *Ambassadors*. From a list of 13 physical, mental and social item banks (see supplement 5), they chose physical function, fatigue, and pain interference as most important. We added depression to this list, since it is strongly associated with the onset of RA [7, 24]. Other secondary outcomes are waist circumference, fat mass (measured by dual-energy X-ray absorptiometry [DEXA]), blood pressure, LDL, HDL, triglycerides, fasting glucose, and ESR.

#### Other outcome measures

Other outcomes related to body composition and metabolism are body weight, height and muscle mass (measured by DEXA). Fifty MSOA patients (30 from the intervention group and 20 from the control group) will be recruited for MRI examination of visceral adipose tissue (VAT), liver fat, and intramuscular fat in the thigh muscle. For the liver and the thigh muscle, the fat content will be determined. In VAT and the thigh muscle, we will characterize the fatty acid distribution based on the peaks in the spectrum representing double and single bonds in the carbon backbone.

Performance-based physical functioning will be measured by grip strength [54] and per get-up-and-go test [55].

Diet (4-7 consequent days digital diary) will be measured using the Dutch ‘Eetmeter’ by ‘Voedingscentrum’ (The Netherlands Nutrition Centre), a validated food diary based on the Dutch food composition database. It is applicable through internet and in the form of an App [56]. Stress is measured using the perceived stress scale (PSS) [57]. In addition, biophysical markers for stress are measured using morning salivary cortisol and heart rate variability (as measured on electrocardiography). Physical activity level will be based on activity data captured by Fitbit Inspire HR wristbands.

We will track metabolic markers such as blood pressure, lipid profile, fasting glucose, and HbA1c and measure pathogenic biomarkers such as RF and ACPA, C-reactive protein, fecal, and oral saliva microbiota 16S composition as well as plasma metabolome changes. Other biomarkers related to dietary intake such as blood markers for folic acid, vitamin B12, calcidiol, hemoglobin, ferritin, leukocytes, thrombocytes, mean corpuscular hemoglobin, creatinine, alanine transaminase, and aspartate transaminase will be measured.

An economic evaluation will be performed to assess the cost-effectiveness of the multidisciplinary lifestyle program compared with usual care for quality-adjusted life-years (QALYs) and the primary outcome measures in all three patient groups. The EQ-5D-5L will be used to measure health-related quality of life. The patients’ EQ-5D-5 L health states will be converted into utility values using the Dutch tariff [58]. QALYs will be estimated by multiplying the patients’ time spent in a certain health state by the respective utility value. Costs will be assessed from a societal (among others informal care, absenteeism costs) and a healthcare perspective [59].

A detailed overview of the measurements is available in supplement 5.

### Sample size calculation

Based on *α* = 0.05 and power (1-β) = 0.80, effects ranged from 0.4 to 1.0 decreases for the DAS28 (estimated from component data of the DAS28 in the case of the trial by Kjeldsen Kragh et al.) with standard deviations (SD) ranging from 0.6–1.0 [12, 14]. The minimal clinically important improvement (MCII) in the DAS28, however, increases with higher baseline values and vice versa [60]. In the range of our inclusion criterion of DAS28 (between 2.6 and 5.1), the MCII is 0.8. Sköldstam et al. reported a difference in the DAS28 of 0.5 in 12 weeks [14]. In our 16-week study, we expect an additional effect on top of that provided by diet alone, due to the combination with exercise and stress management. Based on clinically relevant effect sizes of delta 0.8 with a SD of 1.2 for the DAS28, *α* of 0.05, and *β* of 0.2, we need a sample size of 56.

For OA patients, we found effects ranging from 2.2 to 2.8 for WOMAC pain (OA) and 5.7 to 10 for WOMAC function (OA) and standard deviations (SD) ranging from 0.4 to 0.6 with an exceptional 3.1 for WOMAC pain and 1.4 to 3.1 with an exceptional 10.9 for WOMAC function [15, 61, 62]. Based on these studies, we estimated that a power of 0.80 is feasible with a total of 60–70 subjects (intervention + control group) and planned to aim for 80 subjects for both RA as well as OA patients (total 160 patients) based on a possible dropout rate of approximately 20%.

For patients with ACPA-positive arthralgia, we added a pilot group of 16 participants (*n* = 8 intervention group, *n* = 8 waiting-list control group).

### Statistical analysis

At baseline, half-way (8 weeks) and at the end of the study (16 weeks) outcomes are measured (see supplement 5 for a specification of the measurements).

After conclusion of the randomized controlled trial, data will be cleaned, verified by two researchers, and inspected for errors, inconsistencies, and incomplete information. Outliers will be inspected and corrected if necessary.

Primary outcomes DAS28, RA risk-scores, and WOMAC (all continuous variables) at 8 and 16 weeks in the intervention group will be compared with outcomes at 8 and 16 weeks in the control group, adjusted for baseline scores. Intention-to-treat analyses will be performed, with additional per-protocol analyses. For the analyses, a repeated measures mixed model will be used with random effect for the subjects and fixed effects for group (intervention or control) and baseline values for DAS28, RA risk-score, or WOMAC.

Secondary outcome measures (physical function, fatigue, pain interference, depression, waist circumference, fat mass, blood pressure, LDL, HDL, triglycerides, fasting glucose and ESR) will be analyzed the same way as the primary outcomes. Statistical tests will be limited to primary and secondary outcomes. For other outcomes, descriptive statistics will be used. We will analyze microbiome, remaining metabolic and stress-related outcomes separately and report results in dedicated articles.

In the extension study, the primary and secondary outcomes of the intervention study will become secondary outcomes, and adherence will be the primary outcome. These outcomes are analyzed in the same way as the intervention study, using the baseline, halfway, and end measurements from the 16-week intervention program, as well as 6-, 12-, and 18-month and end (24 month) measurements of the extension study. Intention-to-treat analyses will be performed, with additional per-protocol analyses. Since the extension study is observational, a within-subject analysis will be performed using a repeated measures mixed model with random effect for the subjects and fixed effects for baseline values.

*P* values < 0.05 will be considered to be significant.

## Discussion

The present paradigm of RA treatment is early recognition and prompt suppression of inflammation with drug therapy targeted to achieve and maintain remission or low disease activity [40]. Despite the success of intensified drug therapies, there are still unmet needs. Thirty percent of RA patients do not respond to the preferred medication, suffer from side effects, or become resistant to medication [63]. Also, 69% of RA patients are still limited by pain, fatigue, and reduced mobility [64], and the mortality gap between RA patients and the general population remains despite early aggressive treatment [65]. The unmet needs of RA patients, in combination with the underuse of the present knowledge of environmental risk factors for RA in clinical practice, call for an increased effort to investigate potentially effective non-pharmacologic options in the treatment of these diseases.

On the other hand, while treatment of OA comprises exercise treatment, weight loss, and mental health interventions, these lifestyle-related recommendations lack specificity and consensus [66].

The present protocol is in line with the gaps identified by the EULAR committees. For both RA and OA, more evidence on non-pharmacological interventions is needed with an emphasis on *multidisciplinary* approaches, including physical therapy and exercise, psychological support, and diet [66, 67].

A good example of a lifestyle program in which all these non-pharmacological disciplines are combined is the Ornish Program (USA, covered by most health insurance companies), based on a WFPD, exercise, and stress management. It has been shown to be effective for the treatment of coronary heart disease [28]. Since atherosclerosis and synovitis share pathological features [36, 68] and both RA as well as OA patients are at increased risk for cardiovascular disease, it is worthwhile to not only study the effect of this program on arthritis, but also on the factors that determine the risk of coronary heart disease.

### Strengths

The Plants for Joints diet is developed by registered dietitians and based on the Dutch dietary guidelines. The menus meet recommended daily allowances, except for vitamin B_12_ and D, which are supplemented. Menus are affordable and composed of readily available unprocessed plant-based foods. During the 16-week program, participants learn how to implement this diet into their own lifestyle, allowing for feasible, long-term adherence. The same strength is seen in exercise, stress management, and sleep: implementation of the recommendations is simple and easy to grasp.

We are the first to study a multidisciplinary lifestyle intervention based on more than two disciplines for (increased risk of) RA or MSOA in a randomized, single-blind, controlled trial. The addition of microbiome analyses provides us with the opportunity to study changes in the microbiome as a result of the intervention in relation to changes in disease activity.

We are also the first to study a plant-based diet in RA since the studies by Kjeldsen-Kragh in 1991 and Hafström in 2001 [12, 69], where they concluded that a plant-based diet had several favorable outcomes. Their population, however, was different than current RA patients, who are much more intensively treated and will have lower average baseline disease activity. By keeping medication stable during the trial and tapering it using a standardized scheme, we will be able to provide a realistic picture of the tapering potential.

For OA, we are the first to study a multidisciplinary lifestyle program based on a WFPD. An additional strength is that we have chosen for the particular group of MSOA patients which can be characterized by the systemic profile of unfavorable lifestyle, excessive visceral fat, possible microbiome dysbiosis, low-grade inflammation, and metabolic syndrome. Using MRI, we will study the effects of our intervention on visceral adipose tissue, hepatocellular fat, and intramyocellular fat in the MSOA population in relation to pain, function, and stifness (primary outcome). In this study both the effect of the lifestyle intervention as well as the possible pathophysiological pathways are addressed, thus providing a holistic overview of contributing mechanisms.

### Limitations

A double-blind RCT is not possible in this setting. Also, the many measurements may be a burden for the participants and may interfere with adherence. Furthermore, participation is limited to patients with good literacy and e-health competencies for efficiency reasons. Participation obviously was also limited to patients willing to change their lifestyle. In generalizing findings of our study, this relatively high level of motivation has to be taken into account. In addition, the design leaves out the patient group with low disease activity (DAS28 < 2.6) which is estimated at 40–60% of RA patients [70, 71].

## Conclusion

The results of studies on the effectiveness of an (almost) WFPD for RA and OA patients are promising but have not yet been combined with other lifestyle interventions, despite the potential synergistic effects. Therefore, we wish to study the combined effect (including cost-effectiveness) of a WFPD, exercise, and stress management on disease activity in RA patients, RA risk-score in ACPA-positive arthralgia patients, and pain, function, and stiffness in MSOA patients. Long-term adherence will be studied in a 2-year extension study. We will provide evidence on the level of effectiveness of a multidisciplinary lifestyle intervention, which will help to further specify the role of these non-pharmacological treatments in patients with (an increased risk of) RA and MSOA.

## Trial status

Patient recruitment began in May 2019. The trial is currently underway. Article based on protocol version 7.0 (March 2021). Recruitment of patients is expected to be completed in August 2021.

## Supplementary Information

**Additional file 1: Supplement 1.** SPIRIT checklist

**Additional file 2: Supplement 2.** Plants for Joints Program

**Additional file 3: Supplement 3.** Summary fasting protocol

**Additional file 4: Supplement 4.** Tapering scheme medication

**Additional file 5: Supplement 5.** Detailed overview of measurements

## Data Availability

In addition to the material in the supplements of this article, information on this trial can be obtained upon request. No professional writers were used for this article, and they will be not used for the publication of the results. Trial results will be communicated to participants through e-mail newsletters and webinars or seminars. There are no restrictions posed on publication. The project includes the development of an implementation plan for dissemination among health professionals and possible implementation of the program when it shows to be effective.

## Abbreviations

ACPA: Anti-citrullinated protein antibody
ACR: American College of Rheumatology
DAS28: Disease activity score in 28 joints
DEXA: Dual-energy X-ray absorptiometry
DMARD: Disease-modifying anti-rheumatic drug
en%: Percentage of energy (kilocalories)
ESR: Erythrocyte sedimentation rate
EULAR: European Alliance of Associations for Rheumatology
HDL: High-density lipoprotein
HRV: Heart rate variability
LDL: Low-density lipoprotein
MCII: Minimal clinically important improvement
MRI: Magnetic resonance imaging
MSOA: Metabolic syndrome-associated osteoarthritis
NSAID: Nonsteroidal anti-inflammatory drug
OA: Osteoarthritis
PROMIS®: Patient-Reported Outcomes Measurement Information System
PSS: Perceived stress scale
QALY: Quality-adjusted life-year
RA: Rheumatoid arthritis
RCT: Randomized controlled trial
SFA: Saturated fatty acid
SPIRIT: Standard Protocol Items: Recommendations for Interventional Trials
VAT: Visceral adipose tissue
WFPD: Whole food plant-based diet
WOMAC: Western Ontario and McMaster Universities Osteoarthritis Index
NCEP ATPIII: National Cholesterol Education Program Adult Treatment Panel III

## References

[1] Hu Y, Sparks JA, Malspeis S, Costenbader KH, Hu FB, Karlson EW, Lu B (2017). Long-term dietary quality and risk of developing rheumatoid arthritis in women. Ann Rheum Dis..

[2] de Hair MJ, Landewe RB, van de Sande MG, van Schaardenburg D, van Baarsen LG, Gerlag DM (2013). Smoking and overweight determine the likelihood of developing rheumatoid arthritis. Ann Rheum Dis..

[3] Crowson CS, Matteson EL, Davis JM, Gabriel SE (2013). Contribution of obesity to the rise in incidence of rheumatoid arthritis. Arthritis Care Res (Hoboken).

[4] Turk SA, van Beers-Tas MH, van Schaardenburg D (2014). Prediction of future rheumatoid arthritis. Rheum Dis Clin North Am..

[5] WHO. WHO: Priority Medicines for Europe and the World 2013 Update. Accessed on: March 22 2018.

[6] van Middendorp H, Evers AW (2016). The role of psychological factors in inflammatory rheumatic diseases: From burden to tailored treatment. Best Pract Res Clin Rheumatol..

[7] Lee YC, Agnew-Blais J, Malspeis S, Keyes K, Costenbader K, Kubzansky LD, Roberts AL, Koenen KC, Karlson EW (2016). Post-Traumatic Stress Disorder and Risk for Incident Rheumatoid Arthritis. Arthritis Care Res (Hoboken)..

[8] Glyn-Jones S, Palmer AJ, Agricola R, Price AJ, Vincent TL, Weinans H, Carr AJ (2015). Osteoarthritis. Osteoarthritis. Lancet..

[9] Skoldstam L, Larsson L, Lindstrom FD (1979). Effect of fasting and lactovegetarian diet on rheumatoid arthritis. Scand J Rheumatol..

[10] Uden AM, Trang L, Venizelos N, Palmblad J (1983). Neutrophil functions and clinical performance after total fasting in patients with rheumatoid arthritis. Ann Rheum Dis..

[11] Hafstrom I, Ringertz B, Gyllenhammar H, Palmblad J, Harms-Ringdahl M (1988). Effects of fasting on disease activity, neutrophil function, fatty acid composition, and leukotriene biosynthesis in patients with rheumatoid arthritis. Arthritis Rheum..

[12] Kjeldsen-Kragh J, Haugen M, Borchgrevink CF, Laerum E, Eek M, Mowinkel P (1991). Controlled trial of fasting and one-year vegetarian diet in rheumatoid arthritis. Lancet..

[13] Abendroth A, Michalsen A, Ludtke R, Ruffer A, Musial F, Dobos GJ (2010). Changes of Intestinal Microflora in Patients with Rheumatoid Arthritis during Fasting or a Mediterranean Diet. Forsch Komplementmed..

[14] Skoldstam L, Hagfors L, Johansson G (2003). An experimental study of a Mediterranean diet intervention for patients with rheumatoid arthritis. Ann Rheum Dis..

[15] Messier SP, Mihalko SL, Legault C, Miller GD, Nicklas BJ, DeVita P, Beavers DP, Hunter DJ, Lyles MF, Eckstein F, Williamson JD, Carr JJ, Guermazi A, Loeser RF (2013). Effects of intensive diet and exercise on knee joint loads, inflammation, and clinical outcomes among overweight and obese adults with knee osteoarthritis: the IDEA randomized clinical trial. JAMA..

[16] Fransen M, McConnell S, Harmer AR, Van der Esch M, Simic M, Bennell KL (2015). Exercise for osteoarthritis of the knee: a Cochrane systematic review. Br J Sports Med..

[17] Fransen M, McConnell S, Hernandez-Molina G, Reichenbach S (2014). Exercise for osteoarthritis of the hip. Cochrane Database Syst Rev..

[18] Clinton CM, O'Brien S, Law J, Renier CM, Wendt MR (2015). Whole-foods, plant-based diet alleviates the symptoms of osteoarthritis. Arthritis..

[19] Di Giuseppe D, Bottai M, Askling J, Wolk A (2015). Physical activity and risk of rheumatoid arthritis in women: a population-based prospective study. Arthritis Res Ther..

[20] de Jong Z, Munneke M, Zwinderman AH, Kroon HM, Jansen A, Ronday KH (2003). Is a long-term high-intensity exercise program effective and safe in patients with rheumatoid arthritis? Results of a randomized controlled trial. Arthritis Rheum..

[21] Hurkmans E, van der Giesen FJ, Vliet Vlieland TP, Schoones J, Van den Ende EC (2009). Dynamic exercise programs (aerobic capacity and/or muscle strength training) in patients with rheumatoid arthritis. Cochrane Database Syst Rev..

[22] Evrengul H, Dursunoglu D, Cobankara V, Polat B, Seleci D, Kabukcu S (2004). Heart rate variability in patients with rheumatoid arthritis. Rheumatol Int..

[23] Koopman FA, Tang MW, Vermeij J, de Hair MJ, Choi IY, Vervoordeldonk MJ, Gerlag DM, Karemaker JM, Tak PP (2016). Autonomic Dysfunction Precedes Development of Rheumatoid Arthritis: A Prospective Cohort Study. EBioMedicine..

[24] O'Donovan A, Cohen BE, Seal KH, Bertenthal D, Margaretten M, Nishimi K (2015). Elevated risk for autoimmune disorders in iraq and afghanistan veterans with posttraumatic stress disorder. Biol Psychiatry..

[25] Pradhan EK, Baumgarten M, Langenberg P, Handwerger B, Gilpin AK, Magyari T, Hochberg MC, Berman BM (2007). Effect of Mindfulness-Based Stress Reduction in rheumatoid arthritis patients. Arthritis Rheum..

[26] Ferwerda M, van Beugen S, van Middendorp H, Spillekom-van Koulil S, Donders ART, Visser H, Taal E, Creemers MCW, van Riel PCLM, Evers AWM (2017). A tailored-guided internet-based cognitive-behavioral intervention for patients with rheumatoid arthritis as an adjunct to standard rheumatological care: results of a randomized controlled trial. Pain..

[27] Lee AC, Harvey WF, Price LL, Han X, Driban JB, Wong JB, Chung M, McAlindon TE, Wang C (2017). Mindfulness Is Associated With Treatment Response From Nonpharmacologic Exercise Interventions in Knee Osteoarthritis. Arch Phys Med Rehabil..

[28] Ornish D, Scherwitz LW, Billings JH, Brown SE, Gould KL, Merritt TA, Sparler S, Armstrong WT, Ports TA, Kirkeeide RL, Hogeboom C, Brand RJ (1998). Intensive lifestyle changes for reversal of coronary heart disease. JAMA..

[29] Furman D, Campisi J, Verdin E, Carrera-Bastos P, Targ S, Franceschi C, Ferrucci L, Gilroy DW, Fasano A, Miller GW, Miller AH, Mantovani A, Weyand CM, Barzilai N, Goronzy JJ, Rando TA, Effros RB, Lucia A, Kleinstreuer N, Slavich GM (2019). Chronic inflammation in the etiology of disease across the life span. Nat Med..

[30] Zaiss MM, Joyce Wu HJ, Mauro D, Schett G, Ciccia F (2021). The gut-joint axis in rheumatoid arthritis. Nat Rev Rheumatol..

[31] Jethwa H, Abraham S (2017). The evidence for microbiome manipulation in inflammatory arthritis. Rheumatology (Oxford)..

[32] van Delft MAM, van der Woude D, Toes REM, Trouw LA (2019). Secretory form of RA-associated autoantibodies in serum are mainly of the IgM isotype. Ann Rheum Dis..

[33] Clemente JC, Manasson J, Scher JU (2018). The role of the gut microbiome in systemic inflammatory disease. BMJ..

[34] Puenpatom RA, Victor TW (2009). Increased prevalence of metabolic syndrome in individuals with osteoarthritis: an analysis of NHANES III data. Postgrad Med..

[35] Sekar S, Shafie SR, Prasadam I, Crawford R, Panchal SK, Brown L, Xiao Y (2017). Saturated fatty acids induce development of both MetS and OA in rats. Sci Rep..

[36] Courties A, Sellam J, Berenbaum F (2017). Metabolic syndrome-associated osteoarthritis. Curr Opin Rheumatol..

[37] Hansildaar R, Vedder D, Baniaamam M, Tausche AK, Gerritsen M, Nurmohamed MT (2021). Cardiovascular risk in inflammatory arthritis: rheumatoid arthritis and gout. Lancet Rheumatol..

[38] van de Stadt LA, Witte BI, Bos WH, van Schaardenburg D (2013). A prediction rule for the development of arthritis in seropositive arthralgia patients. Ann Rheum Dis..

[39] Chan AW, Tetzlaff JM, Altman DG, Laupacis A, Gotzsche PC, Krleza-Jeric K (2013). SPIRIT 2013 statement: defining standard protocol items for clinical trials. Ann Intern Med..

[40] Aletaha D, Neogi T, Silman AJ, Funovits J, Felson DT, Bingham CO (2010). 2010 rheumatoid arthritis classification criteria: an American College of Rheumatology/European League Against Rheumatism collaborative initiative. Ann Rheum Dis..

[41] Fransen J, van Riel PL (2006). DAS remission cut points. Clin Exp Rheumatol.

[42] Altman R, Alarcon G, Appelrouth D, Bloch D, Borenstein D, Brandt K (1991). The American College of Rheumatology criteria for the classification and reporting of osteoarthritis of the hip. Arthritis Rheum..

[43] Altman R, Asch E, Bloch D, Bole G, Borenstein D, Brandt K, Christy W, Cooke TD, Greenwald R, Hochberg M, Howell D, Kaplan D, Koopman W, Longley S, Mankin H, McShane DJ, Medsger T, Meenan R, Mikkelsen W, Moskowitz R, Murphy W, Rothschild B, Segal M, Sokoloff L, Wolfe F (1986). Development of criteria for the classification and reporting of osteoarthritis. Classification of osteoarthritis of the knee. Diagnostic and Therapeutic Criteria Committee of the American Rheumatism Association. Arthritis Rheum..

[44] 3rd Report of the NCEP Expert Panel on High Blood Cholesterol in Adults. Circulation. 2002;106(25):3143–421. 10.1161/circ.106.25.3143. 12485966

[45] Barnard N, Gloede L, Cohen J, Jenkins DJ, Turner-McGrievy G, Green AA (2009). A low-fat vegan diet elicits greater macronutr changes. J Am Diet Assoc..

[46] Melina V, Craig W, Levin S (2016). Position of the Academy: Vegetarian Diets. J Acad Nutr Diet..

[47] Gezondheidsraad. Beweegrichtlijnen 2017. Accessed on: March 29 2018. Link: https://www.gezondheidsraad.nl/nl/taak-werkwijze/werkterrein/preventie/beweegrichtlijnen-2017.

[48] de Brouwer SJ, Kraaimaat FW, Sweep FC, Donders RT, Eijsbouts A, van Koulil S (2011). Psychophysiological responses to stress in patients with RA. PLoS One..

[49] van Riel PL (1992). Provisional guidelines for measuring disease activity in clinical trials on rheumatoid arthritis. Br J Rheumatol..

[50] Bellamy N, Buchanan WW, Goldsmith CH, Campbell J, Stitt LW (1988). Validation study of WOMAC: a health status instrument for measuring clinically important patient relevant outcomes to antirheumatic drug therapy in patients with osteoarthritis of the hip or knee. J Rheumatol..

[51] Roorda LD, Jones CA, Waltz M, Lankhorst GJ, Bouter LM, van der Eijken JW, Willems WJ, Heyligers IC, Voaklander DC, Kelly KD, Suarez-Almazor ME (2004). Satisfactory cross cultural equivalence of the Dutch WOMAC in patients with hip osteoarthritis waiting for arthroplasty. Ann Rheum Dis..

[52] Ornish D, Lin J, Chan JM, Epel E, Kemp C, Weidner G, Marlin R, Frenda SJ, Magbanua MJM, Daubenmier J, Estay I, Hills NK, Chainani-Wu N, Carroll PR, Blackburn EH (2013). Effect of comprehensive lifestyle changes on telomerase activity and telomere length in men with biopsy-proven low-risk prostate cancer: 5-year follow-up of a descriptive pilot study. Lancet Oncol..

[53] Dutch-Flemish PROMIS. Link: http://www.dutchflemishpromis.nl/index.php.

[54] Roberts HC, Denison HJ, Martin HJ, Patel HP, Syddall H, Cooper C, Sayer AA (2011). A review of the measurement of grip strength in clinical and epidemiological studies: towards a standardised approach. Age Ageing..

[55] Piva SR, Fitzgerald GK, Irrgang JJ, Bouzubar F, Starz TW (2004). Get up and go test in patients with knee osteoarthritis. Arch Phys Med Rehabil..

[56] Ocke M, Dinnissen C, Stafleu A, de Vries J, van Rossum C. Relative Validity of MijnEetmeter: A Food Diary App for Self-Monitoring of Dietary Intake. Nutrients. 2021;13(4). 10.3390/nu13041135 PMC8066644 33808209

[57] Cohen S, Kamarck T, Mermelstein R (1983). A global measure of perceived stress. J Health Soc Behav..

[58] Versteegh M (2016). Impact on the Incremental Cost-Effectiveness Ratio of Using Alternatives to EQ-5D in a Markov Model for Multiple Sclerosis. Pharmacoeconomics..

[59] Kanters TA, Bouwmans CAM, van der Linden N, Tan SS, Hakkaart-van RL (2017). Update of the Dutch manual for costing studies in health care. PLoS One..

[60] Aletaha D, Funovits J, Ward MM, Smolen JS, Kvien TK (2009). Perception of improvement in patients with rheumatoid arthritis varies with disease activity levels at baseline. Arthritis Rheum..

[61] Messier SP, Loeser RF, Miller GD, Morgan TM, Rejeski WJ, Sevick MA, Ettinger WH, Pahor M, Williamson JD (2004). Exercise and dietary weight loss in overweight and obese older adults with knee osteoarthritis: the Arthritis, Diet, and Activity Promotion Trial. Arthritis Rheum..

[62] Miller GD, Nicklas BJ, Davis C, Loeser RF, Lenchik L, Messier SP (2006). Intensive weight loss program improves physical function in older obese adults with knee osteoarthritis. Obesity (Silver Spring)..

[63] Hazlewood G, Barnabe C, Tomlinson G, Marshall D, Devoe DJ, Bombardier C. MTX monother & MTX comb ther with trad & biol DMARDs for RA. CDSR. 2016:CD010227. 10.1002/14651858.CD010227.pub2 PMC7087436 27571502

[64] Sloot R, Flinterman L, Heins M, Lafeber M, Boeije H, Poos R (2016). Reumatische aandoeningen in Nederland - Ervaringen en kengetallen.

[65] Holmqvist M, Ljung L, Askling J (2018). Mortality following new-onset RA. Ann Rheum Dis..

[66] Fernandes L, Hagen KB, Bijlsma JW, Andreassen O, Christensen P, Conaghan PG, Doherty M, Geenen R, Hammond A, Kjeken I, Lohmander LS, Lund H, Mallen CD, Nava T, Oliver S, Pavelka K, Pitsillidou I, da Silva JA, de la Torre J, Zanoli G, Vliet Vlieland TP, European League Against Rheumatism (EULAR) (2013). EULAR recommendations for the non-pharmacological core management of hip and knee osteoarthritis. Ann Rheum Dis..

[67] Combe B, Landewe R, Daien CI, Hua C, Aletaha D, Alvaro-Gracia JM (2017). 2016 update of the EULAR recommendations for the management of early arthritis. Ann Rheum Dis..

[68] Sattar N, McCarey DW, Capell H, McInnes IB (2003). Explaining how "high-grade" systemic inflammation accelerates vascular risk in rheumatoid arthritis. Circulation..

[69] Hafstrom I, Ringertz B, Spangberg A, von Zweigbergk L, Brannemark S, Nylander I (2001). A vegan diet free of gluten improves the signs and symptoms of rheumatoid arthritis: the effects on arthritis correlate with a reduction in antibodies to food antigens. Rheumatology (Oxford)..

[70] Versteeg GA, Steunebrink LMM, Vonkeman HE, Ten Klooster PM, van der Bijl AE, van de Laar M (2018). Long-term disease and patient-reported outcomes of a continuous treat-to-target approach in patients with early rheumatoid arthritis in daily clinical practice. Clin Rheumatol..

[71] Einarsson JT, Willim M, Ernestam S, Saxne T, Geborek P, Kapetanovic MC (2019). Prevalence of sustained remission in rheumatoid arthritis: impact of criteria sets and disease duration, a Nationwide Study in Sweden. Rheumatology (Oxford)..

