# Supplementary material for: The effect of a multidisciplinary lifestyle program for patients with rheumatoid arthritis, an increased risk for rheumatoid arthritis or with metabolic syndrome-associated osteoarthritis: the “Plants for Joints” randomized controlled trial protocol
Source: Trials. 2021 Oct 18;22:715. doi: 10.1186/s13063-021-05682-y (PMC8524903; doi:10.1186/s13063-021-05682-y)
Supplement: Supplementary file 2 — Additional file 2: Supplement 2. Plants for Joints Program [file 13063_2021_5682_MOESM2_ESM.docx]

**Supplement 2: Plants for Joints Lifestyle Program**

After recruitment patients are pre-screened (by telephone) and receive detailed information on the program and the required measurements for the scientific research. The first visit (>1 w later) starts with the definitive screening and informed consent and concludes with a personal intake with the dietician, baseline measurements and randomization. In the first month of the program all participants will determine personal objectives, abilities and limitations regarding exercise during a personal consultation with a physiotherapist specialized in joint diseases.

During the program subjects meet 10 times in groups of 6-12 people. Participants bring a partner to the first meeting (cooking class). During all meetings (2-3 hours/meeting) subjects receive theoretical and practical training, based on protocols tested in previous studies on the following topics.

**Whole foods plant-based diet**

Recommendations are based on the Dutch Dietary Guidelines and protocols by a.o. Ornish and Barnard. Subjects receive fully elaborated week plans, dietary guidelines, recipes, and a cooking class led by a chef specialized in healthy, whole food plant-based cooking. Week plans are made by registered dieticians and are in line with Dutch dietary guidelines and recommended daily allowances. Subjects also receive vitamin B12 and vitamin D supplementation.

**Exercise**

Exercise recommendations are based on the Dutch physical activity guidelines 2017 and personal objectives. Subjects are introduced to different forms of moderately intense exercise such as brisk walking, cycling, yoga, and muscle strengthening exercises. Group practice is focused on a sustainable increase in physical activity, fun and group cohesion. Patients will be motivated to integrate exercise in daily activities and verify possibilities in their direct neighbourhood. Group practices are led by personal trainers experienced in patients with chronic diseases.

**Stress management & sleep**

Stress management education and exercises are based on protocols by Ornish and de Brouwer. Subjects receive education on the effects of stress and sleep on health outcomes, as well as guided practice and home exercises (supported by tools, such as audio/Apps) on relaxation techniques (including progressive, cue controlled, and differential relaxation), breathing and visualization exercises and coaching on sleep.

Group supervisors promote cohesion, amongst others by facilitating a digital platform to share experience, meal pictures, questions and progress. Friendly reminders are send to enhance adherence to the daily physical and relaxation exercise regime. A total of 2-3 representatives per discipline are involved: dieticians, occupational therapists specialized in mindfulness based stress reduction and sleep and physio- or exercise therapists.

Most group meetings start with a *roundtable discussion* focused on sharing successes, helping group members answering questions (how do you …?) covering subjects as cooking tips, exercise tips, stress management tips and motivational issues.

| **Meeting** | **Description** |
| --- | --- |
| 1 | Cooking class with partner (or someone else who can serve as 'support' during program). |
| 2 | Theory: impact of lifestyle on health, whole food plant-based diet, stress, exercise.  Introduction stress management, relaxation exercise (bodyscan) and mindful eating/tasting (observation exercise). |
| 3 | Nutrition product knowledge (e.g. reading labels): what are processed foods, rich in fat, sugar and salt? How can I recognize them? What are my healthy options and alternatives?  Why we should be physically active, the downwards spiral (including an exercise on why one is less physically active, awareness), including brisk walking or other easy exercise.  Homework: practicing relaxation during the day, mindful eating and other relaxation exercises (https://www.reade.nl/ontspanning, VGZ Mindfulness App). |
| 4 | Group meeting; Roundtable discussion (short version, only questions)  Assessing own abilities, body awareness and ‘exercise test’ (to be repeated in week 13)  Relaxation & exertion, parasympathetic & sympathetic nervous systems (with exercise to get awareness of these systems) and breathing exercises |

| 5 | Group meeting; Roundtable discussion  Theory physical activity (including physical activity guidelines 2017 by the NL Health Council)  Physical activity versus exercise and discussion on motivation to stop  Homework: relaxation- and breathing exercise (https://www.reade.nl/ontspanning) |
| --- | --- |
| 6 | Group meeting; Roundtable discussion  Exercise: homework  Sleep training (basic education and 'sounder sleep exercise') |
| 7 | Group meeting; Analysing the 'group menu'. What do we eat for breakfast, lunch, diner and in between meals?  The importance of flexibility, body balance, yoga, pilates  Homework: relaxation exercise (https://www.reade.nl/ontspanning) and choosing a favourite exercise |
| 8 | Group meeting; Roundtable discussion  Potluck: participants take their most successful recipe  Exercise: homework  Experience and theory on stress management: how to deal with thoughts (to allow them or not to allow them) and inventory of questions and needs for the 10^th^ group meeting |
| 9 | Group meeting; Roundtable discussion and summary presentation of nutrition theory of meetings 2 and 3.  Basic forms of exercise theory and practical application (coordination, strength, velocity, flexibility and endurance)  Homework: relaxation exercise (https://www.reade.nl/ontspanning) |
| 13 | Group meeting; Sharing successes, future plans and perpetuation of the group.  Roundtable discussion (homework preparation: what makes me (not) maintain my improvements in daily practice?) with celebration of successes  Repetition of most important lessons on diet  Assessing own abilities, body awareness and ‘exercise test’ (repetition week 4)  Questions and planning for the time to come. Long relaxation exercise as conclusion |
