# Supplementary material for: The effect of a multidisciplinary lifestyle program for patients with rheumatoid arthritis, an increased risk for rheumatoid arthritis or with metabolic syndrome-associated osteoarthritis: the “Plants for Joints” randomized controlled trial protocol
Source: Trials. 2021 Oct 18;22:715. doi: 10.1186/s13063-021-05682-y (PMC8524903; doi:10.1186/s13063-021-05682-y)
Supplement: Supplementary file 3 — Additional file 3: Supplement 3. Summary fasting protocol [file 13063_2021_5682_MOESM3_ESM.docx]

**Supplement 3: Summary green fasting protocol of Plants for Joints**

Five studies conducted between 1979 and 2010, showed favorable outcomes for 7-10 days of fasting in a clinical setting for patients with rheumatoid arthritis. See table 1.

Table 1: overview of studies on fasting for patients with rheumatoid arthritis.

| Author & year | N | Design | Intervention | Results |
| --- | --- | --- | --- | --- |
| Sköldstam et al  1979 | I: 16  C: 10 | RCT | 7-10 days 200 kcal/day juice fasting | Decreased pain, stiffness, Ritchie Articular Index (RAI), *finger ring size* and *clinical 6 joint score*, ESR (not significant) and lower use of analgesics. |
| Udén et al  1983 | 13 | Cross over | 7 days 0 kcal | Baseline- vs end measurement, improved RAI, ESR, stiffness. |
| Hafström et al  1988 | 14 | Observational | 7 days 0 kcal | Baseline- vs end measurement improved ESR, stiffness, and *articular index* (tender/ swollen joints). |
| Kjeldsen-Kragh et al 1991 | I: 27  C: 26 | RCT | 7-10 days 200-300 kcal/day juice fasting (no fruits) | After 1 month: improved  RAI, ESR, CRP, number of tender and swollen joints, pain and stiffness. |
| Abendroth 2010 | 22 | Clinical trial | 7 days Buchinger 800 kcal/day | Baseline- vs end measurement DAS28 5.7±0.9 vs 4.1±1.3. |

Fifteen target group patients (*Ambassadors*) are involved in the development of the lifestyle program and the analyses. These experts experienced changes in their own health status with diet adaptations, exercise and/or use of stress management techniques.

The Plants for Joints ambassadors recommended to integrate a fasting protocol in the Plants for Joints lifestyle program. Since Plants for Joints is an outpatient program and the fasting programs listed in table 1 were all inpatient programs, we decided to develop a lighter version of a fasting protocol.

The Plants for Joints fasting protocol is suited for all participants, unless otherwise advised by the involved researchers, registered dietitians or medical doctors.

Standard duration is 4 days, although participants can extend the fasting to a maximum of 6 days.

**Fasting protocol**

Preparation:

- Stop using coffee 2 days before start (tea is allowed during fasting).
- Stop alcohol consumption at least 1 week before start.
- As it concerns a short period, additional supplementation is not necessary. Use of multivitamins during fasting is allowed.

Day 1

Eat approximately half of what is normally consumed in a day. I.e., half portions of the sample menu.

Day 2-3 (to be extended to a maximum of 4 days)

During the core fasting period, participants are recommended to eat only vegetables (preferably green vegetables) and to avoid intake of sugars (no fruit), fat (no oil or fat containing foods such as avocado, nuts and seeds), starch (e.g. rice, bread) and foods containing proteins (e.g. legumes, soy products).

Ginger and lemon juice can be used as seasoning. It is recommended to eat vegetables in any form: soups with low salt vegetable broth, boiled or steamed green vegetables or smoothies. Juices of green leafy vegetables are also suitable, maximum 1 liter (4 large glasses).

Day 4

Eat approximately half of what is normally consumed in a day. I.e., half portions of the sample weekly menu.
