# Supplementary material for: The effect of a multidisciplinary lifestyle program for patients with rheumatoid arthritis, an increased risk for rheumatoid arthritis or with metabolic syndrome-associated osteoarthritis: the “Plants for Joints” randomized controlled trial protocol
Source: Trials. 2021 Oct 18;22:715. doi: 10.1186/s13063-021-05682-y (PMC8524903; doi:10.1186/s13063-021-05682-y)
Supplement: Supplementary file 4 — Additional file 4: Supplement 4. Tapering scheme medication [file 13063_2021_5682_MOESM4_ESM.docx]

**Supplement 4: Tapering scheme medication**

Rheumatoid arthritis (RA) patients will visit the rheumatology nurse every 3 months. If in minimal disease activity (DAS<2.6) at any time point, medication will be tapered according to the below schedule. If disease activity increases after tapering, medication will again be increased back to the previous step. Any deviation from the schedule below will be recorded, but otherwise have no consequences.

*DMARD Monotherapy*

1. Methotrexate (MTX) monotherapy will be adapted according to the following schedule:
2. The MTX dose will be decreased with 7,5 mg/week per 3 months for dosages between 20-25 mg/week to 10 mg/week followed by step (d) when DAS remains <2.6.
3. For MTX dose of 17,5 or 15 mg/week, doses will be decreased to 10 mg/week at once followed by step (d) when DAS remains <2.6.
4. An MTX dose of 12,5 mg/week will be decreased to 7,5 mg mg/week at once followed by step (d) when DAS remains <2.6.
5. For MTX dosages of 10 or 7,5 mg/week, dosages will be decreased to 5 mg/week at once followed by step (e) when DAS remains <2.6.
6. MTX can be stopped when the remaining dosage is 5 mg/week and DAS remains <2.6.
7. Leflunomide dose will be decreased with 10 mg/day per 3 months (and stopped, when DAS remains <2.6).
8. Sulfasalazine dose will be decreased with 500 mg/day per 3 months (and stopped, when DAS remains <2.6).
9. Hydroxychloroquine dose will be decreased with 200 mg/d per 3 months (and stopped, when DAS remains <2.6).
10. Prednisone dose ≤7,5 mg/day will be decreased with 2,5 mg/d in a 7-week schedule per 3 months, doses ≥10 mg/day can be decreased at once with 2,5 mg/day per 3 months (and stopped, when DAS remains <2.6).
11. Biological disease-modifying anti-rheumatic drugs (bDMARDs) can be tapered according to the Reade tapering protocol, doubling the interval two times per 3 months and then stopping when DAS remains <2.6.

*Combination therapy including bDMARD*

The bDMARD is first tapered to stop, followed by a decrease in conventional synthetic DMARDs (csDMARDs) in parallel steps, if more than one type used.

*Combination therapy including csDMARD*

1. MTX and prednisone: first prednisone is decreased to stop, then MTX.
2. MTX, prednisone, sulfasalazine and hydroxychloroquine: first prednisone is decreased to stop, then sulfasalazine, then hydroxychloroquine and then MTX.
3. With all (other) combination therapy, MTX is tapered as last, except in case of side effects, then MTX is first lowered to a more tolerable dosage.

Additional pain therapy (e.g. paracetamol, tramadol, NSAIDs or morphine) can be tapered according to patient preferences.

Osteoarthritis (OA) patients are contacted by the research nurse every 3 months (telephone at 3 and 9 months; visit at 6 and 12 months). Pain therapy is recorded, and patients are stimulated by the research nurse to lower pain therapy (50% every 3 months) when perceived pain is low.
