# Supplementary material for: The effect of a multidisciplinary lifestyle program for patients with rheumatoid arthritis, an increased risk for rheumatoid arthritis or with metabolic syndrome-associated osteoarthritis: the “Plants for Joints” randomized controlled trial protocol
Source: Trials. 2021 Oct 18;22:715. doi: 10.1186/s13063-021-05682-y (PMC8524903; doi:10.1186/s13063-021-05682-y)
Supplement: Supplementary file 5 — Additional file 5: Supplement 5. Detailed overview of measurements [file 13063_2021_5682_MOESM5_ESM.docx]

# Supplement 5: Detailed overview of measurements

|  | **Unit** | | **Source** | | **16-week RCT** | | | | **Extension study** | |
| --- | --- | --- | --- | --- | --- | --- | --- | --- | --- | --- |
|  |  |  |  |  | **Baseline** | | **Half-way** | **End** | **6 m/18 m** | **12 m/24 m** |
| **Primary outcomes** |  | |  | |  | |  |  |  |  |
| RCT 1: DAS-28 (RA) | - | | Clinical checklist | | x | | x | x | x | x |
| RCT 2: WOMAC (OA) | - | | WOMAC | | x | | x | x | x | x |
| RCT 3: RA-risk score (arthralgia) | - | | Clinical checklist | | x | | x | x | x | x |
| Extension study: Adherence | - | | Formula | | x | | x | x | x | x |
|  |  | |  | |  | |  |  |  |  |
| **Secondary outcomes** |  | |  | |  | |  |  |  |  |
| Self reported physical/mental/social health^[[1]](#endnote-1)^ | - | | PROMIS® | | x | | x | x | x | x |
| Metabolic syndrome: |  | |  | |  | |  |  |  |  |
| Waist circumference | cm | | Tape | | x | | x | x | x | x |
| Fat mass | %body weight | | DEXA | | x | |  | x |  | x |
| Blood pressure | mmHg | | BP meter | | x | | x | x | x | x |
| Lipid profile: |  | |  | |  | |  |  |  |  |
| LDL | mmol/l | | Blood | | x | | x | x | x | x |
| HDL | mmol/l | | Blood | | x | | x | x | x | x |
| Triglycerides | mmol/l | | Blood | | x | | x | x | x | x |
| Fasting glucose | mmol/l | | Blood | | x | | x | x | x | x |
| Inflammation: |  | |  | |  | |  |  |  |  |
| Erythrocyte sedimentation rate (ESR) | mmol/h | | Blood | | x | | x | x | x | x |
|  |  | |  | |  | |  |  |  |  |
| **Other outcomes** |  | |  | |  | |  |  |  |  |
| Metabolism, muscle & fat mass: |  | |  | |  | |  |  |  |  |
| Body weight | kg, clothes no shoes | | Scale | | x | | x | x | x | x |
| Body height | cm | | Stadiometer | | x | |  |  |  |  |
|  | **Unit** | | **Source** | | **16-week RCT** | | | | **Extension study** | |
|  |  |  |  |  | **Baseline** | | **Half-way** | **End** | **6 m/18 m** | **12 m/24 m** |
| BMI | kg/m2 | | Calculation | | x | | x | x | x | x |
| HbA1c | mmol/mol | | Blood | | x | | x | x | x | x |
| Muscle mass | %body weight | | DEXA | | x | |  | x |  | x |
| Visceral adipose tissue | ratio’s | | MRS (50 subj) | | x | |  | x |  |  |
| Liver fat content | % of fat mass | | MRS (50 subj) | | x | |  | x |  |  |
| Intramuscular fat mass in the thigh muscle | % of fat mass | | MRS (50 subj) | | x | |  | x |  |  |
| Types of fat in thigh muscle | ratio's | | MRI (50 subj) | | x | |  | x |  |  |
| Lipid profile: |  | |  | |  | |  |  |  |  |
| n-3 fatty acids | mmol/l | | Blood | | x | | x | x | x | x |
| n-6 fatty acids | mmol/l | | Blood | | x | | x | x | x | x |
| Microbiome: |  | |  | |  | |  |  |  |  |
| Microbiota (feces, not measured at 24 m) | | CFU/g | | Feces | | x | x | x |  | x |
| Metabolome (plasma) | | molecules | | Blood | | x | x | x | x | x |
| Microbiota (saliva) | | CFU/g | | Saliva | | x |  | x |  |  |
| Metabolome (urine) | | molecules | | Urine | | x | x | x | x | x |
| Stress & quality of life: |  | |  | |  | |  |  |  |  |
| Self reported physical/mental/social health^[[2]](#endnote-2)^ | - | | PROMIS® | | x | | x | x | x | x |
| Stress |  | | PSS | |  | |  |  |  |  |
| Salivary cortisol (not measured at 24 m) | | nmol/l | | Saliva | | x |  | x |  | x |
| Heart rate variability (not measured at 24 m) |  | | ECG | | x | |  | x |  | x |
| Economic evaluation: |  | |  | |  | |  |  |  |  |
| EQ-5D-5L | - | | Questionnaire | | x | | x | x | x | x |
| Cost-diary (use of health care) | - | | Questionnaire | | x | | x | x | x | x |
| Function: |  | |  | |  | |  |  |  |  |
|  | **Unit** | | **Source** | | **16-week RCT** | | | | **Extension study** | |
|  |  |  |  |  | **Baseline** | | **Half-way** | **End** | **6 m/18 m** | **12 m/24 m** |
| Hand grip strength | kg/force | | Dynamometer | | x | |  | x |  | x |
| Function (get-up-and-go-test) | sec | | GUG-test | | x | |  | x |  | x |
| Physical activity level (PAL) | coefficient/BMR | | Pedometer | | x | | x | x | x | x |
| Pathogenic/disease related: |  | |  | |  | |  |  |  |  |
| C-reactive protein | mg/l | | Blood | | x | | x | x | x | x |
| IgM-RF (RA & arthralgia) | U/ml | | Blood | | x | | x | x | x | x |
| Anti-CCP (RA & arthralgia) | U/ml | | Blood | | x | | x | x | x | x |
| B-cell receptor clones (arthralgia) | % | | Blood | | x | |  | x |  | x |
| CTX-1 | pg/ml | | Blood | | x | |  | x |  | x |
| Diet (7-day diary): |  | | Eetmeter App | | x | | x | x | x | x |
| Energy intake (kcal/day) | kcal/day | | - | | x | | x | x | x | x |
| Macronutrient intake |  | | Eetmeter App | | x | | x | x | x | x |
| Protein | g | | - | | x | | x | x | x | x |
| Fats | en% | | - | | x | | x | x | x | x |
| Carbohydrates | en% | | - | | x | | x | x | x | x |
| Fibres | g | | - | | x | | x | x | x | x |
| Alcohol | g | | - | | x | | x | x | x | x |
| Micronutrient intake |  | | Eetmeter App | | x | | x | x | x | x |
| Calcium | mg | | - | | x | | x | x | x | x |
| Iodine | μg | | - | | x | | x | x | x | x |
| Iron | mg | | - | | x | | x | x | x | x |
| Magnesium | mg | | - | | x | | x | x | x | x |
| Potassium | mg | | - | | x | | x | x | x | x |
| Selenium | μg | | - | | x | | x | x | x | x |
| Zinc | mg | | - | | x | | x | x | x | x |
| Vitamin A | μg | | - | | x | | x | x | x | x |
| Vitamin B1/thiamin | mg | | - | | x | | x | x | x | x |
|  | **Unit** | | **Source** | | **16-week RCT** | | | | **Extension study** | |
|  |  |  |  |  | **Baseline** | | **Half-way** | **End** | **6 m/18 m** | **12 m/24 m** |
| Vitamin B2/riboflavin | mg | | - | | x | | x | x | x | x |
| Vitamin B3/niacin | mg | | - | | x | | x | x | x | x |
| Vitamin B6/pyridoxin | mg | | - | | x | | x | x | x | x |
| Vitamin B11/folic acid | μg | | - | | x | | x | x | x | x |
| Vitamin B12/cobalamin | μg | | - | | x | | x | x | x | x |
| Vitamin C/ascoric acid | mg | | - | | x | | x | x | x | x |
| Vitamin D/cholecalciferol | μg | | - | | x | | x | x | x | x |
| Vitamin E/tocopherol | mg | | - | | x | | x | x | x | x |
| Folic acid in plasma | nmol/l | | Blood | | x | | x | x | x | x |
| Free vitamin B12 (holotranscobalamin) | pmol/l | | Blood | | x | | x | x | x | x |
| Calcidiol (vitamin D/25-OH) | nmol/l | | Blood | | x | | x | x | x | x |
| Other: |  | |  | |  | |  |  |  |  |
| General questions (a.o. smoking) | - | | Questionnaire | | x | | x | x | x | x |
| Haemoglobin | mmol/l | | Blood | | x | | x | x | x | x |
| Leukocytes | nr cells/l | | Blood | | x | | x | x | x | x |
| Trombocytes | nr platelets/l | | Blood | | x | | x | x | x | x |
| Mean corpuscular haemoglobin (MCV) | | femtolitre | | Blood | | x | x | x | x | x |
| Creatinine | | μmol/l | | Blood | | x | x | x | x | x |
| Alanine transaminase (ALAT) | | U/l | | Blood | | x | x | x | x | x |
| Aspartate transaminase (ASAT) | | U/l | | Blood | | x | x | x | x | x |
| Ferritin | | μg/l | | Blood | | x | x | x | x | x |
| Stored serum | | - | | Blood | | x | x | x | x | x |

**Abbreviations** ACPA: Anti-citrullinated protein antibody, DAS28: Disease activity score in 28 joints, DEXA: Dual-energy X-ray absorptiometry, en%: Percentage of energy (kilocalories), ESR: Erythrocyte sedimentation rate, HDL: High density lipoprotein, HRV: Heart rate variability, IgM: Immunoglobulin M, LDL: Low density lipoprotein, MRI: Magnetic resonance imaging, MRS: Magnetic resonance spectroscopy, OA: Osteoarthritis, PROMIS®: Patient-Reported Outcomes Measurement Information System, PSS: Perceived stress scale, RA: Rheumatoid arthritis, RCT: Randomized controlled trial, SFA: Saturated fatty acid, UFA: Unsaturated fatty acid, VAT: Visceral adipose tissue, WOMAC: Western Ontario and McMaster Universities Osteoarthritis Index

1. PROMIS® questionnaires and computer adaptive tests (CATs). Item banks: physical function, fatigue, pain interference and depression are secondary outcomes. Pain intensity, sleep disturbance, sleep related impairment, depression, anxiety, instrumental social support, emotional support, practical support, social isolation, ability to participate in social roles are measured and categorized in other outcomes (not tested for main publication on primary outcomes). [↑](#endnote-ref-1)
2. PROMIS® questionnaires and computer adaptive tests (CATs). Item banks: physical function, fatigue, pain interference and depression are secondary outcomes. Pain intensity, sleep disturbance, sleep related impairment, depression, anxiety, instrumental social support, emotional support, practical support, social isolation, ability to participate in social roles are measured and categorized in other outcomes (not tested for main publication on primary outcomes). [↑](#endnote-ref-2)
